# Editorial: Cutaneous Leishmaniasis: Exploring Pathogenesis and Immunomodulatory Approaches

**DOI:** 10.3389/fcimb.2021.839851

**Published:** 2022-01-18

**Authors:** Wander Rogério Pavanelli, Izabel Galhardo Demarchi

**Affiliations:** ^1^ Departamento de Ciências Patológicas, Centro de Ciências da Saúde. Universidade Estadual de Londrina, Londrina, Brazil; ^2^ Departamento de Análises Clínicas, Centro de Ciências da Saúde. Universidade Federal de Santa Catarina, Florianópolis, Brazil

**Keywords:** *Leishmania*, parasite, antiprotozoal agents, immunomodulatory agent, immunomodulation effect, tegumentary leishmaniasis

Leishmaniases comprises a class of diseases that are caused by *Leishmania* protozoa. They are considered ancient enemies that emerged as successful parasites that invade both invertebrate and vertebrate hosts millions of years ago. Cutaneous leishmaniasis (CL) has affected ~1 million people worldwide, manifesting as different clinical forms (World Health Organization, 2021a; World Health Organization, 2021b). This form of trypanosomiasis is considered a neglected tropical disease, but it has also been reported in economically privileged countries (e.g., the United States; World Health Organization, 2021c). The complex and various life cycles of *Leishmania* and their ability to inhabit a wide range of hosts and diverse ecological niches have been associated with their persistence and expansion. In mammals, its pathogenesis consists of complex interactions between the parasite and the host’s immune system, resulting in persistence of the parasite with different clinical manifestations and mechanisms of elimination (Kaye and Scott, 2011). Considering the variability of *Leishmania* species and hosts, the mammals’ immune response orchestrates the success or failure of killing the pathogen (Kaye et al., 2020). Drug treatment is clearly essential, but the usual anti-leishmaniasis drugs can have severe adverse effects and toxicity, resulting in therapeutic failure or patient dropout from therapy. Thus, new immunomodulatory therapies need to be developed to successfully treat CL. The scientific community, global agencies, and governments have made efforts to develop strategies to eliminate leishmaniasis. Such research has provided critical insights into the mechanisms that influence the full spectrum of leishmaniasis, including its pathogenesis, diagnosis, and treatment.

Volpedo et al. reviewed the immunopathogenesis of CL and post-Kala-azar dermal leishmaniasis. They synthesized on balance between Th1 and Th2 responses for infection control. The exaggerated polarization of Th cells appears to be responsible for severe disease pathology. Gomez et al. explored early leukocyte responses in ex vivo models of healing and non-healing human *Leishmania (Viannia) panamensis* infections. The elucidation of immunological mechanisms will undoubtedly contribute to strategies to develop the next generation of therapeutics and vaccines for CL.

Pentavalent antimonials remain the first-choice therapy for CL because no effective human vaccines are currently available. Meglumine antimoniate is the most common drug that is used for CL treatment. In a recent systematic review by Pinar et al. (2020), moderate evidence was found for cure rates and adverse effects when intramuscular meglumine antimoniate was used for American CL. Thus, drug combinations have been proposed as alternative therapies to treat TL (Berbert et al., 2018). In a brief research report, a pilot randomized clinical trial was conducted using oral miltefosine and pentavalent antimonials that were combined with pentoxifylline to treat American TL.The authors observed similar cure rates and a lower risk of adverse effects (Martins et al.). Exploring new approaches on leishmaniasis drugs, Craig et al. explored new approaches to develop anti-leishmaniasis drugs and found that thermoresponsive copolymer nanoparticles improve the bioavailability of retrograde inhibitors *in vitro*. Compound encapsulation in copolymer was a viable strategy to dramatically increase the bioavailability and efficacy of anti-*Leishmania* compounds.

Although some studies have reported promising results, problems and limitations with various treatments remain. To explain therapeutic failures, resistance, and susceptibility, Fernández et al. showed human neutrophil activation ex vivo that influenced tolerance to meglumine antimoniate and the susceptibility of clinical strains of *L. (V.) panamensis*. They found lower reactive oxygen species production and higher CD62L and CD66b expression on cells that were infected with tolerant/resistant *L. (V.) panamensis* compared with cells that were infected with drug-sensitive strains.

Among tegumentary leishmaniasis, *Leishmania (Viannia) braziliensis* is the most prevalent parasite that is identified in infected mammals. This species is responsible for mucocutaneous leishmaniasis in Latin America (World Health Organization, 2021b). Souza et al. investigated the role of miR-548d-3p in *L. (V.) braziliensis* infection. The parasite appears to interfere with chemokine production by modulating miRNAs, consequently affecting inflammatory processes that are essential for lesion resolution. They suggested that miR-548d-3p may be a prognostic marker for TL and a host-directed therapeutic target. dos Santos et al. explored the intestines as a new target organ of chronic *L. (V.) braziliensis* infection in hamsters, highlighting a possible target for future studies to understand susceptibility and resistance. Animal models are frequently used to understand the pathogenesis of leishmaniasis and develop new therapeutic strategies (Cabral et al., 2021). Tomiotto-Pellissier et al. reported that arginase-1 and macrophages at lesion sites influence susceptibility to *Leishmania amazonensis* in mice. Their findings on protective immunity against *L. amazonensis* suggest new approaches to treat and prevent this disease.

A brief research report by de Lima et al. arelates high anti-Leishmania IgG antibody levels with the severity of mucosal leishmaniasis in humans. The reduction of antibody production could indicate treatment success in most patients. The authors suggested that these tests can be applied to assess therapeutic response.

Although no vaccine is currently available for CL and considering that vaccines are effective primary strategies to prevent infectious diseases, the in silico screening and rational selection of potential candidates on a large scale have been used as research tools prior to *in vitro* and *in vivo* evaluations (Flórez et al.). In the field of pharmaceutical sciences, these authors described Leishmania spp. epitopes in humans that were naturally resistant to leishmaniasis, working toward a synthetic vaccine.

The reports on cutaneous leishmaniasis in this Research Topic highlight areas that demand further investigation. Cutaneous leishmaniasis remains a global problem, requiring effective diagnostic measures and treatments. Considering the global health situation post-COVID-19 and neglected tropical diseases (Tilli et al., 2021), we strongly recommend further support for leishmaniasis research, based on promising results that have been generated to date.

## Author Contributions

All authors listed have made a substantial, direct, and intellectual contribution to the work and approved it for publication.

## Conflict of Interest

The authors declare that the research was conducted in the absence of any commercial or financial relationships that could be construed as a potential conflict of interest.

## Publisher’s Note

All claims expressed in this article are solely those of the authors and do not necessarily represent those of their affiliated organizations, or those of the publisher, the editors and the reviewers. Any product that may be evaluated in this article, or claim that may be made by its manufacturer, is not guaranteed or endorsed by the publisher.

## References

[B1] Berbert T. R. N. de Mello T. F. P. Nassif P. W. Mota C. A. Silveira A. V. Duarte G. C.. (2018). Pentavalent Antimonials Combined With Other Therapeutic Alternatives for the Treatment of Cutaneous and Mucocutaneous Leishmaniasis: A Systematic Review. Dermatol. Res. Pract. 2018, 9014726. doi: 10.1155/2018/9014726 30675152 PMC6323433

[B2] Cabral F. V. Souza T. H. D. S. Sellera F. P. Fontes A. Ribeiro M. S. (2021). Towards Effective Cutaneous Leishmaniasis Treatment With Light-Based Technologies. A Systematic Review and Meta-Analysis of Preclinical Studies. J. Photochem. Photobiol. B. 221, 112236. doi: 10.1016/j.jphotobiol.2021.112236 34090038

[B3] Kaye P. M. Cruz I. Picado A. Van Bocxlaer K. Croft S. L. (2020). Leishmaniasis Immunopathology-Impact on Design and Use of Vaccines, Diagnostics and Drugs. Semin. Immunopathol. 42 (3), 247–264. doi: 10.1007/s00281-020-00788-y 32152715

[B4] Kaye P. Scott P. (2011). Leishmaniasis: Complexity at the Host-Pathogen Interface. Nat. Rev. Microbiol. 9 (8), 604–615. doi: 10.1038/nrmicro2608 21747391

[B5] Pinart M. Rueda J. R. Romero G. A. Pinzón-Flórez C. E. Osorio-Arango K. Maia-Elkhoury A. N. S.. (2020). Interventions for American Cutaneous and Mucocutaneous Leishmaniasis. Cochrane Database Syst. Rev. 8 (8), CD004834. doi: 10.1002/14651858.CD004834.pub3 32853410 PMC8094931

[B6] Tilli M. Olliaro P. Gobbi F. Bisoffi Z. Bartoloni A. Zammarchi L. (2021). Neglected Tropical Diseases in non-Endemic Countries in the Era of COVID-19 Pandemic: The Great Forgotten. J. Travel Med. 28 (1), taaa179. doi: 10.1093/jtm/taaa179 32970143 PMC7543559

[B7] World Health Organization, WHO. (2021a). Cutaneous Leishmaniasis. Available at: https://www.who.int/leishmaniasis/cutaneous_leishmaniasis/en/#:~:text=Cutaneous%20leishmaniasis%20is%20the%20most,the%20face%2C%20arms%20and%20legs.&text=When%20the%20ulcers%20heal%2C%20they,cause%20of%20serious%20social%20prejudice.

[B8] World Health Organization, WHO. (2021b). Mucocutaneous Leishmaniasis. Available at: https://www.who.int/leishmaniasis/mucocutaneous_leishmaniasis/en/#:~:text=In%20mucocutaneous%20leishmaniasis%2C%20the%20lesions,being%20rejected%20by%20the%20community.

[B9] World Health Organization, WHO. (2021c). Control of Neglected Tropical Diseases. Available at: https://www.who.int/teams/control-of-neglected-tropical-diseases/neglected-zoonotic-diseases.

